# Six dilemmas for stakeholders inherently affecting data sharing during a zoonotic (re-)emerging infectious disease outbreak response

**DOI:** 10.1186/s12879-024-09054-0

**Published:** 2024-02-12

**Authors:** Martine Y. van Roode, Carolina dos S. Ribeiro, Elmoubasher Farag, Mohamed Nour, Aya Moustafa, Minahil Ahmed, George Haringhuizen, Marion P.G. Koopmans, Linda H.M. van de Burgwal

**Affiliations:** 1 Department of Viroscience, Erasmus University Medical Center (Erasmus MC), Dr. Molewaterplein 40, 3015 GD, Rotterdam, The Netherlands; 2 Center for Infectious Disease Control, The Netherlands National Institute for Public Health and the Environment (RIVM), Bilthoven, The Netherlands; 3 Vrije Universiteit Amsterdam (VU Amsterdam), Faculty of Science, Athena Institute for Research On Innovation and Communication in Health and Life Sciences, Amsterdam, The Netherlands; 4 Department of Health Protection & Communicable Diseases, Ministry of Public Health, Doha, Qatar; 5 Pandemic and Disaster Preparedness Center (PDPC), Rotterdam, The Netherlands

**Keywords:** Data sharing, Infectious disease, Outbreak response, Zoonoses, One Health

## Abstract

**Background:**

Timely access to outbreak related data, particularly in the early events of a spillover, is important to support evidence based control measures in response to outbreaks of zoonotic Emerging Infectious Diseases (EID). Yet, this is impeded by several barriers that need to be understood to promote timely sharing of data. Using the MERS epidemic as a model for a zoonotic EID outbreak, this study sought to provide an in-depth understanding of data sharing practices.

**Methods:**

Semi-structured interviews with 25 experts were conducted, along with Focus Group Discussions with 15 additional experts. A root-cause analysis was performed to examine the causal relationships between barriers. Enablers were mapped to the root-cause analysis to understand their influence on the barriers. Finally, root causes were placed in context of core dilemmas identified from the qualitative analysis.

**Findings:**

Eight barriers to data sharing were identified, related to collaboration, technical preparedness, regulations, and (conflict of) interests, and placed in the context of six dilemmas inherent to the multi-stakeholder collaboration required for a zoonotic outbreak response. Fourteen identified enablers showed the willingness of stakeholders to overcome or circumvent these barriers, but also indicated the inherent trial and error nature of implementing such enablers.

**Interpretation:**

Addressing the barriers requires solutions that must consider the complexity and interconnectedness of the root causes underlying them, and should consider the distinct scopes and interests of the different stakeholders. Insights provided by this study can be used to encourage data sharing practices for future outbreaks

**Funding:**

Wellcome Trust and UK Aid; EU-H2020 Societal Challenges (grant agreement no. 643476), Nederlandse Organisatie voor Wetenschappelijk Onderzoek (VI.Veni.201S.044)

**Supplementary Information:**

The online version contains supplementary material available at 10.1186/s12879-024-09054-0.

## Research in context

### Evidence before this study

The swift and transparent sharing of outbreak related data, particularly in the early events of a spillover when outbreak control may still be feasible, is a crucial pillar of a zoonotic outbreak response. Several studies to date have identified barriers to data sharing; yet these barriers persist. While issues with e.g. ownership have been documented, a comprehensive and in-depth analysis of barriers to data sharing, and the root causes underlying them, in a zoonotic outbreak response, requiring national, international, and global stakeholder collaboration across sectors and disciplines, is lacking; as are factors that enabled data sharing.

### Added value of this study

This comprehensive study deciphered the complexity of data sharing in a multi-stakeholder environment during a zoonotic EID outbreak response, and particularly in the early events of a spillover. While improvements to strengthen global health security at the human-animal interface through cross-sectoral collaboration and activities have been made since the MERS epidemic, the root causes deciphered in this study further indicate areas of improvement necessary for supporting such a One Health approach. Moreover, one of the main issues brought forward during the current review process of the proposed amendments to the International Health Regulations (IHR), is the need for timely sharing of pathogen information, specifically at the human-animal interface. Therefore, some of the, so far unpublished, data on barriers and enablers to data sharing in the early stages of the MERS epidemic provided by this study, could provide helpful to that discussion.

### Implications of all the available sources

The current organization of stakeholders and the alignment of outbreak investigation and response activities no longer meet society’s needs in terms of swiftness and effectiveness in a zoonotic outbreak response, demanding an enhanced level of multi-stakeholder collaboration, particularly at the human-animal interface. This means finding a way to overcome the barriers to data sharing and stakeholders’ dilemmas, inherent to such collaboration. It underlines the importance of a necessary dialogue between and amongst relevant stakeholders to achieve a better informed, and more sophisticated decision on the improvement of data sharing practices, through reciprocity and incentive mechanisms, and considering the inherent trial and error nature thereof. This will constitute a crucial pillar in preparedness for future zoonotic EID outbreaks, in view of the current threat of zoonotic spill overs, as posed by e.g. bird flu.

## Introduction

There is an inevitable need for rapid and open data sharing during zoonotic emerging infectious disease (EID) outbreaks, particularly in the early events of a spillover when outbreak control may still be feasible [1, 2]. Rapid sharing of epidemiological, clinical and research data, including pathogen sequence data, is crucial to provide real-time guidance for public health response actions and to quickly identify and address knowledge gaps during outbreaks [3–5].

Yet, a multitude of interrelated barriers delay or hamper timely data sharing [6–8], hindering efficient public health responses, and ultimately outbreak control. These barriers can be complex in nature and difficult to circumvent, especially during outbreaks that involve multiple sectors and countries. This is often the case when dealing with (re-)emerging zoonotic disease outbreaks, when transmission routes may be complex and cross state lines, collaboration across sectors and/or disciplines is essential, and outbreak response almost by definition has economic and social consequences [9]. Furthermore, data sharing practices may vary depending on priorities and perspectives from stakeholders.

The Middle East respiratory syndrome (MERS) epidemic, with its onset in 2012, was caused by a newly emerging Coronavirus (MERS-CoV), for which camels were identified as asymptomatic reservoir animals [10, 11]. Due to continuous new introductions of MERS-CoV into the human population, MERS-CoV constituted a constant threat for public health. Therefore, it was used in this study as a model for a zoonotic EID as it offered a real-life experience where a wide array of stakeholders, with competing interests, different priorities, attitudes, and ownership issues were involved in the outbreak investigation and public health response [12, 13]. All these aspects need to be studied to understand practices concerning the accessibility and timely sharing of data as a key component of preparedness for future zoonotic EID outbreaks.

## Methods

This study used qualitative methods to allow for identification and in-depth investigation of barriers and enablers for outbreak related data sharing [8]. The emphasis of this case study was placed on understanding the sharing of technical data at the animal – human interface (Table 1), through an in-depth review of the outbreak investigation done in Qatar from 2012 until 2019, together with eliciting perspectives from stakeholders on the response in the wider region of the Arabian Peninsula.

**Table 1:** Scope of study

Types of data
*Epidemiological investigation and surveillance*: tracking of cases and contacts, outbreak investigation, including identification of sources and transmission modes;
*Clinical research*: research involving the systematic observations of, data collection from, diagnostic or intervention(s) on multiple or individual cases;
*Laboratory research*: research involving all activities concerning laboratory outbreak response and research, including the sharing of microbial genetic resources, i.e. strains and genetic sequence data from pathogens and related metadata, samples, assays, protocols, and experiences
**Levels of data sharing (**representing the operating levels of stakeholders**)**
*National*, where data is shared among stakeholders within Qatar and is used to monitor population health, target response, and resource allocation;
*Regional*, where data is shared among countries in the same region, or a group of countries with a collaborating institute or organization;
*International*, where data is shared among countries and organizations outside the region, including research institutes;
*Global*, where data is shared among international agencies, and inter-agency levels, and is used to estimate the global burden of disease and to contain emerging global health threats

## Data collection

The selection of key stakeholders, who were found relevant to the response, was based on purposive sampling of an a priori defined population, with a role and/or involvement in the understanding, responding to and controlling of the MERS outbreak in Qatar, operating at the global, international, regional and national level (Table 1), and representing supranational organizations, public health or animal health institutes, and academic research institutes. A broad population of potential stakeholders was identified in a literature study [14], in which a timeline of events that occurred through the MERS epidemic was constructed. Furthermore, stakeholders at the national level were identified through the network of the Qatari Supreme Council of Health and Ministry of Public Health, and the Qatari Ministry of Municipality and Environment. While the focus of this case study was the outbreak investigation done in Qatar, for eliciting perspectives from stakeholders on the response in the wider region of the Arabian Peninsula, senior officials of the relevant national authority of three countries in the region with a role and/or involvement in the understanding, responding and controlling of MERS in the Gulf region were invited to participate in this study, in addition to stakeholders representing regional offices of supranational organizations. After applying the stakeholder inclusion criteria (Table 2), a subset of this population remained, consisting of 70 stakeholders, who were invited to participate in this study.

**Table 2:** Stakeholder inclusion (left) and exclusion (right) criteria

Inclusion criteria	Exclusion criteria
Work of stakeholder is/was related to MERS public health response or related research during the outbreaks	Stakeholder is unavailable during time frame of data collection of this case study
Stakeholder has/had authority to make (a) decision(s) on data sharing or a key role in, or key influence on, data sharing within their operating level(s) (national, regional, international, global)	Stakeholder feels he/she has not much to say or has insufficient knowledge about the topic of the case study and/or refers to others for answering to case study interview questions
Stakeholder is/was collaborating and sharing data with stakeholders from Qatar	Stakeholder is part of the case study team
Stakeholder is/was involved in laboratory or public health research that directly or indirectly shaped the public health response in Qatar	
Stakeholder was involved in crucial phases of the outbreak response or related research	

In total, 70 stakeholders were invited to participate. The invitation included operational and ethical arrangements (e.g. confidentiality, informed consent procedure), as well as a list of examples of data types and most-commonly used data sharing mechanisms to provide clarity on the focus of this study (Supplementary Material 3).

### Semi-structured interviews

Stakeholders were interviewed face-to-face or by phone. Using a standardized interview topic list (Supplementary Material 4), the semi-structured interviews lasted between 36 to 78 min. The interviews were recorded, transcribed, and pseudo-anonymized.

### Expert group discussions

Aimed to bring about a common understanding of enablers and barriers, and recommendations on actions to improve data sharing, a workshop involving the national stakeholders in Qatar was conducted in November 2018 to reflect on the interim study results and the (anonymized) overall viewpoints and experiences of other stakeholders who had participated in the semi-structured interviews. Participants were divided into three expert groups where discussion was moderated (1.5 h per group), based on their sector of involvement: representing the human health and animal health sector, supporting the One Health Joint field investigation team; representing academia, supporting research; representing laboratories, supporting the field investigations.

## Data analysis

**Fig. 1:**
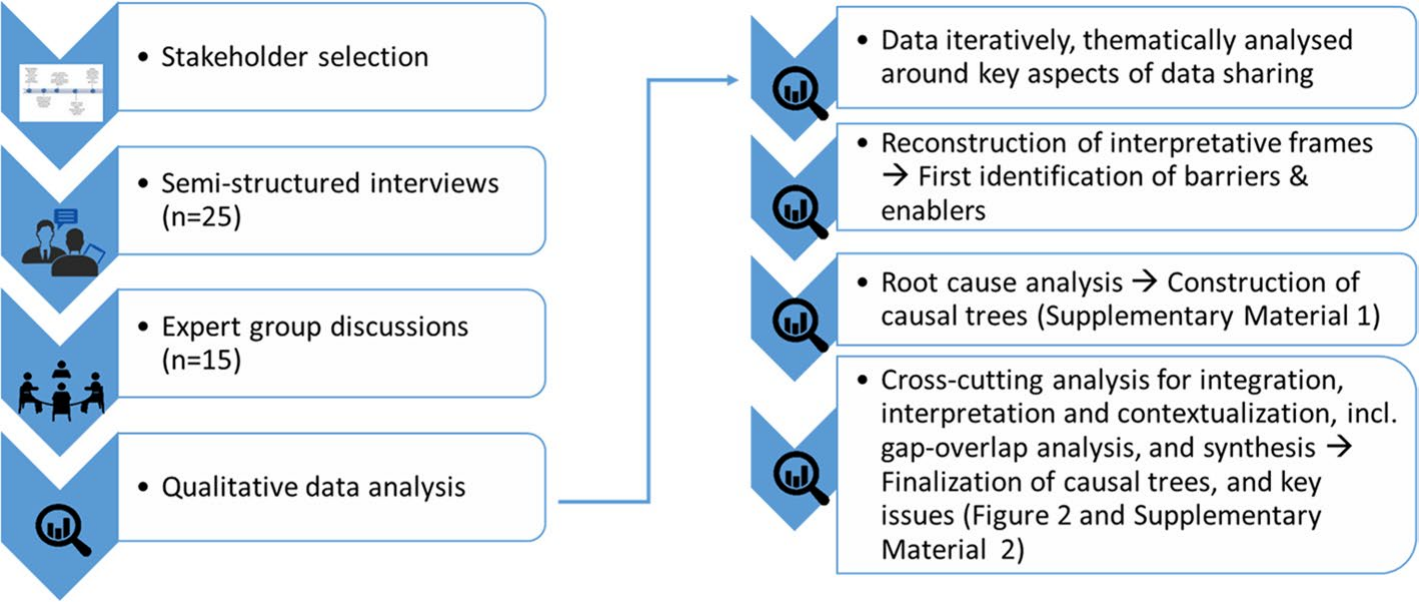
Overview of the methods (left), with details on the qualitative data analysis steps, leading to the results described in this paper (right)

The data collected were thematically analyzed according to standard practices in qualitative research [15, 16] (Fig. 1). The researchers followed five predetermined steps: familiarization, identifying the thematic framework, data indexing, data charting, data mapping and data interpretation [15]. For thematic analysis of the interviews, an a priori established coding framework—informed by the case study aims and introduced into the interviews via the standard interview topic list—was used, which was complemented during the analysis by emergent issues or recurrence of particular experiences by the respondents [16]. Using the reconstruction of interpretative frames technique, the researchers familiarized themselves with the stakeholders’ perceptions on and interests in data sharing during the MERS epidemic, including barriers and enablers that were experienced [17], followed by a root-cause analysis to examine the causal relationships between barriers. The researchers initially analyzed the data separately, followed by discussion of the preliminary results in the team for construction of the causal trees. Enablers were mapped to the root-cause analysis to understand their influence on the barriers. For integration, interpretation and contextualization of the results, iterative discussions were held amongst the researchers, as well as a gap-overlap analysis to highlight and analyze where findings reinforce and/or contradict each other. Consequently, the final causal trees were constructed that were used to conclude the key issues in data sharing, and finally, root causes were placed in context of core dilemmas identified from the qualitative analysis, for which a discussion was held amongst the researchers.

### Role of funding source

The funders were not directly involved in defining the study design; data collection, analysis and interpretation; writing of the report; and decision to submit the paper for publication.

## Results

In total, 40 key stakeholders participated (response rate 57%; Table 3). These stakeholders provided combined expertise that was considered to be relevant for three out of four defined levels of data sharing: global, international and national. The regional level was excluded as the number of participants was low (*n* = 2), and not deemed sufficient representative of the regional level. Stakeholders were from Europe, Australia, the U.S., and Qatar.

**Table 3:**
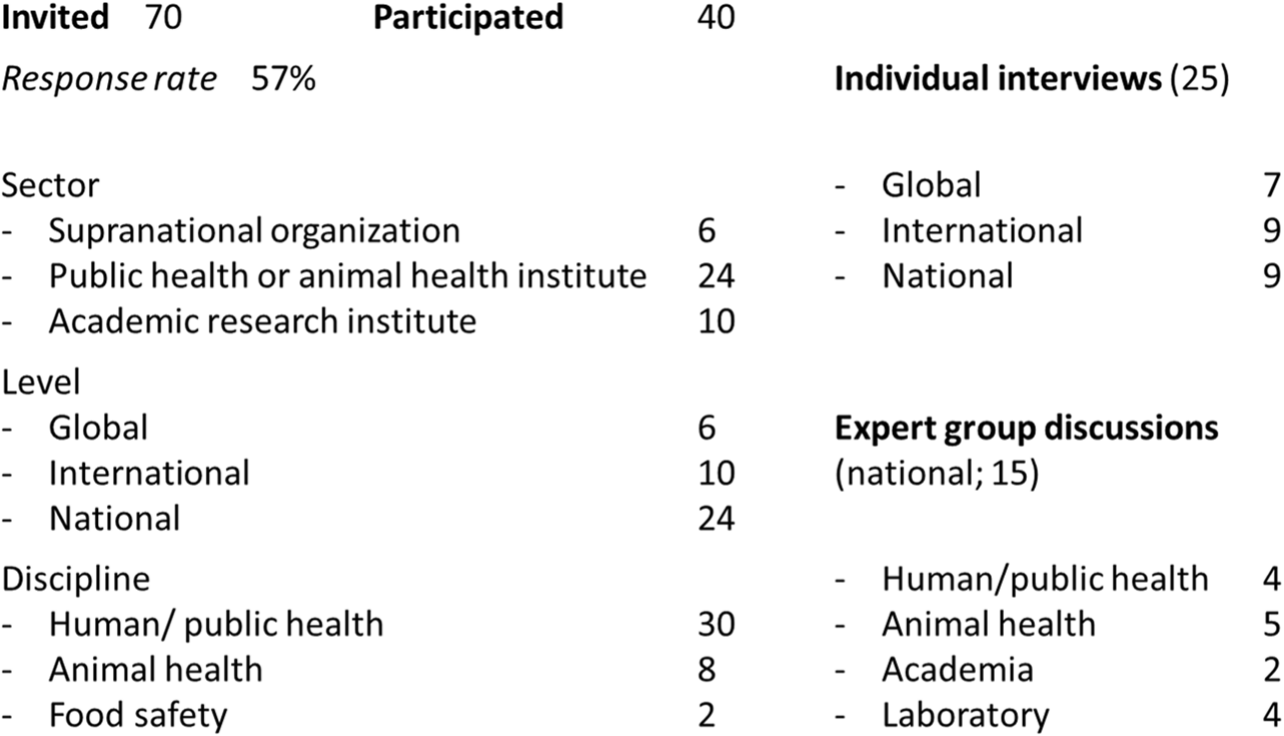
Stakeholders represented different sectors, operating levels, and disciplines (left), and were individually interviewed (right, top) or participated in the expert group discussions (bottom). As stakeholders can be active across two sectors or disciplines, their representation in this table reflects their main area of expertise

## Barriers to data sharing

The root cause analysis, shown in Supplementary Material 1(a-e), revealed eight distinct barriers that hampered or delayed data sharing during the MERS epidemic (Table 4). Causal factors underlying these barriers overlapped, indicating the interconnected and systemic nature of these barriers.

**Table 4:** Barriers to data sharing

*Barriers that hampered or delayed data sharing during the MERS epidemic*
1. Suboptimal One Health collaboration between public health and animal health stakeholders
2. Suboptimal collaboration between public health and lab research partners
3. Difficulties in local collection, handling and processing of samples and data
4. Delays in formal notification of human cases – including associated clinical and epidemiological data – and infected animals with MERS-CoV
5. Delayed formal clearance to access and share (sensitive) data
6. Difficulties to timely ship and import samples potentially containing infectious material
7. Prioritization of scientific publications over sharing of data relevant for outbreak investigation and response
8. Establishing and adhering to ownership agreements delay and limit sharing of data

‘Suboptimal One Health collaboration between public health and animal health stakeholders’ (*barrier 1,* Supplementary Material 1a) was most notably characterized by the delayed engagement of the animal sector, owing to the delayed recognition of camels as a potential reservoir, coupled with an initial hesitancy from camel owners and farmers in allowing the sampling of camels. In this barrier, fragmentation was seen in the responsibilities and priorities of stakeholders across disciplines and sectors. Another root cause was fragmentation in the costs and benefits between disciplines and sectors—the animal sector, and responsible ministry, needed to invest in what was primarily perceived as a human health problem, since camels were asymptomatic.

‘Suboptimal collaboration between public health and lab research partners’ (*barrier 2,* Supplementary Material 1a), expressed the challenges by partners from these different sectors to quickly access each other’s data. Root causes were attributed to the lack of common mechanisms and platforms for sharing of outbreak related data, and insufficient investment in local laboratory capacity.

For both barriers, the level of coordinated and structural collaboration between stakeholders that was necessary to deal with the complexity of such an outbreak, exceeded the level of, mostly disease-specific and ad hoc, collaboration that was in place. Both barriers were causally influenced by fragmented mandates and responsibilities of stakeholders, resulting in delayed stakeholders’ sharing of data, collaboration and alignment of response activities across disciplines and sectors. While barrier 1 mainly affected data sharing, and subsequently the outbreak response, by stakeholders at the national and global levels, barrier 2 mainly involved and affected national stakeholders and response actions.

‘Difficulties in local collection, handling and processing of samples and data’ (*barrier 3,* Supplementary Material 1b) characterized the technical capacity and capability to collect, generate and share data as part of a routine, complex outbreak response. Root causes were, amongst others, the delay in locally implemented standardized protocols and guiding tools for sample collection and handling; coupled with the inherent complexity in outbreak investigations of unknown zoonotic EIDs. This barrier was also influenced by the fragmentation issues in stakeholder collaborations (barriers 1 and 2), such as the delayed engagement of the animal sector, which caused delays in the collection of epidemiological data and samples from camels. This was further complicated by the delayed guidance on investigation and case definitions for reporting MERS-CoV in animals, causing a late establishment and activation of a One Health surveillance system. Although this barrier mainly concerned stakeholders at the national level, yet data sharing with stakeholders at all levels was affected.

‘Delays in formal notification of human cases – including associated clinical and epidemiological data – and infected animals with MERS-CoV’ (*barrier 4,* Supplementary Material 1c) were caused by a range of issues. Some related to disputes over the current formal notification process, such as the timeliness to report cases, which could be delayed as each of the affected countries had concerns about its interests and the potential consequences of sharing its data (e.g. on trade, tourism, etc.). Other issues were attributed to the delayed establishment of an active surveillance system for animals, as well as technical difficulties (stated in barriers 1 and 3). Barrier 4 mainly concerned national and global stakeholders, although international stakeholders were also affected, especially when they wanted to publish data that were yet to be notified formally. ‘Delayed formal clearance to access and share (sensitive) data’ (*barrier 5,* Supplementary Material 1d) stemmed from the institutional policies, procedures and processes to which the stakeholders have to adhere before sharing data. It showed that the perceived negative consequences of data sharing had an adverse impact on decisions to share data. This mainly concerned the national stakeholders, but the delayed data sharing affected all other stakeholders. The barrier ‘Difficulties to timely ship and import samples potentially containing infectious material’ (*barrier 6,* Supplementary Material 1d) depicted the complexity in involved regulations of shipping samples (‘Infected substances’) internationally. This further complicated data sharing between national and international stakeholders.

Finally, ‘prioritization of scientific publications over sharing of data relevant for outbreak investigation and response’ (*barrier 7,* Supplementary Material 1e), and stakeholders’ conflict of interests in assigning ownership rights, as reflected in barrier ‘Establishing and adhering to ownership agreements delay and limit sharing of data’ (*barrier 8,* Supplementary Material 1e), led to delays in data sharing. Although the national and international stakeholders were mainly affected by the last two barriers, the root causes revealed that the lack of globally agreed and standardized mechanisms for data sharing during outbreaks was no less important cause.

## Enablers to data sharing

Fourteen distinct enablers were identified (Table 5). These were mentioned to have either motivated or facilitated data sharing during the MERS epidemic. They were classified into three categories: indisputable (*n* = 7), situational/contextual (*n* = 4), and tentative (*n* = 3) enablers. The *indisputable enablers* were the ones found to have consistently facilitated data sharing practices; while the *situational enablers* were the ones found to have the ability to influence data sharing depending on the situation or the context in which they occur (having either the capacity to facilitate or hamper data sharing). The *tentative enablers* were subject of some debate. The interaction of the enablers with the barriers was visualized in the root-cause analysis (Supplementary Material 1a-d).

**Table 5:** Enablers to data sharing. Numbers in this table refer to the numbers shown in Supplementary Material 1

**Indisputable Enablers**, facilitating data sharing
1. Political commitment from national authorities for transparency in and endorsement for data sharing: • at the national level to share data across public and animal health stakeholders to stimulate (One-Health) collaboration. For instance, through the establishment of a dedicated One-Health Joint field investigation team unifying the Ministries responsible for public and animal health (1a); • stimulating and engaging in collaboration and data sharing with international and global stakeholders (1b)
2. Pre-existing collaborations and networks facilitated the response and helped to further build trust necessary for data sharing between stakeholders, and included networks involving: • The public health and animal health stakeholders that was established during previous outbreaks at national level (2a); • The public health and partners of research laboratories that was established during previous outbreaks or as part of ongoing research at national level (2b); • The national and international stakeholders through the WHO network (e.g. reference centers) (2c); • at the international level established through previous research activities and consortia (2d)
3. Confidential meetings that allowed for direct, rapid, and confidential sharing of state-of-the-art information and data through open conversations between stakeholders, facilitating and/or building forward on a certain level of trust. Such meetings were relevant: • when data sharing and data access in collaborations were not (yet) formalized (3a); • to inform authorities and provide guidance on how to handle safety concerns, like shipments (3b); • to share novel scientific and outbreak-related data that could potentially be crucial for the outbreak response but were not yet published (3c)
4. Reciprocity and bilateral data sharing ensured mutual benefits, fairness, and respectful collaborations. One example is sharing data by the national authorities with international partners in return for the capacity building and the technical expertise, like training and diagnostics
5. Setting good examples through showing the benefits of data sharing: • Countries with political commitment for data sharing—of which the national authorities were timely reporting cases and notifying under the IHR, and shared data openly—getting rapid technical assistance in return for data sharing particularly during the first period of an unfolding outbreak of an emerging pathogen, set a positive example followed by some other countries (5a); • sharing potentially crucial research/outbreak data before publication in scientific journals in the interest of rapid public health response, for example during confidential meetings, helped building trust in consolidating the technical partnerships and set a good example that was followed by other scientists (5b)
6. Expedited publication process: fast-track reviews of manuscripts by scientific journals, and acceptance of pre-published data in publications seemed to have enabled sharing of crucial outbreak related information without jeopardizing scientific reward
7. Development of diagnostic kits and materials with non-infectious abilities, ensured biosafety and helped overcome safety concerns related to the import of potentially infectious materials and allowed distributing of kits to (countries with) labs compatible with their biosafety levels, empowering local diagnostic capacity
**Situational enablers**, depending on the situation/ context facilitating or hampering data sharing
8. Rigor in public risk communication: Careful, unified public messaging in communicating risks: • the carefulness not to adopt a solo-authority rhetoric blaming a particular thing, group, or an activity of cultural value to the community where an outbreak is unfolding, seemed to have enabled continued collaboration and data sharing. During MERS-CoV, public health authorities worked together with the Animal Health Department to release the carefully designed and jointly cleared public messages as camels had an economic, cultural, and social importance in the local community. This practice allowed building trust with the animal sector to facilitate the subsequent investigation and research (animal sampling and collection, and sharing of related data) (8a); • however, the ambiguous language that was used (by the public health stakeholders) in the public communication reflecting the scientific uncertainty on the potential role of camels as reservoir animals may have delayed the recognition of the animal source. This inherited insufficient engagement of the animal sector and subsequently caused poor sense of urgency to initiate quick and adequate response to this zoonotic threat (8b)
9. Establishment of ownership agreements: • memorandum of Understandings (MoUs) and MTAs brought legal clarity for sharing parties. This clarity of rights and obligations seemed to have enhanced the readiness to sharing data (9a); • however, when negotiations were long, and terms were strict, delays in sharing practices ensued (9b)
10. Clear (hierarchical) coordination and communication chains in data sharing: • MERS-CoV seemed to have stimulated the discussion of accountability in data sharing. Consequently, concerned officials were clear with whom outside their departments to share data, and who was responsible within their institutions to approve such data sharing (10a); • however, if built upon hierarchical processes of approval, this led to delays, and limited flexibility and empowerment of stakeholders, in data sharing (10b)
11. Pressure for data sharing from international stakeholders: • promoted change in attitude for data sharing from certain stakeholders, as this pressure was in favor of the technical officials to convince their leaders to respond to the international requests of sharing data, using the expected mutual benefits as a solid argument (11a); • but this is subject to the relationship of the national authority with the other international/ regional bodies and countries. It has led to a defensive position of some stakeholders to protect their interests (11b)
**Tentative enablers**, unstable enablers pertaining to their implementation
12. The tripartite WHO, WOAH (former OIE), FAO collaboration motivated and created a platform for One-Health collaboration and data sharing; but this initiative could have been improved by enhancing coordination, guidance, and proactivity during the first epidemic phases to avoid “lateness”, which seemed to have originated from conflicts of interests and mandate discussions
13. Formal notification channels (WHO/WOAH) improved sharing of case- and outbreak-related data; but could have been improved in clarity and detail in the guidance for notification, enforcement, and—for the WOAH channel—timely implementation
14. Informal notification under the IHR (WHO) helped confidential sharing of suspected cases and getting support from WHO in the investigations and reporting; creating more awareness about this possibility and how to use it could have further improved data sharing

### Six rooted and systemic dilemmas for stakeholders concerning data sharing

**Fig. 2:**
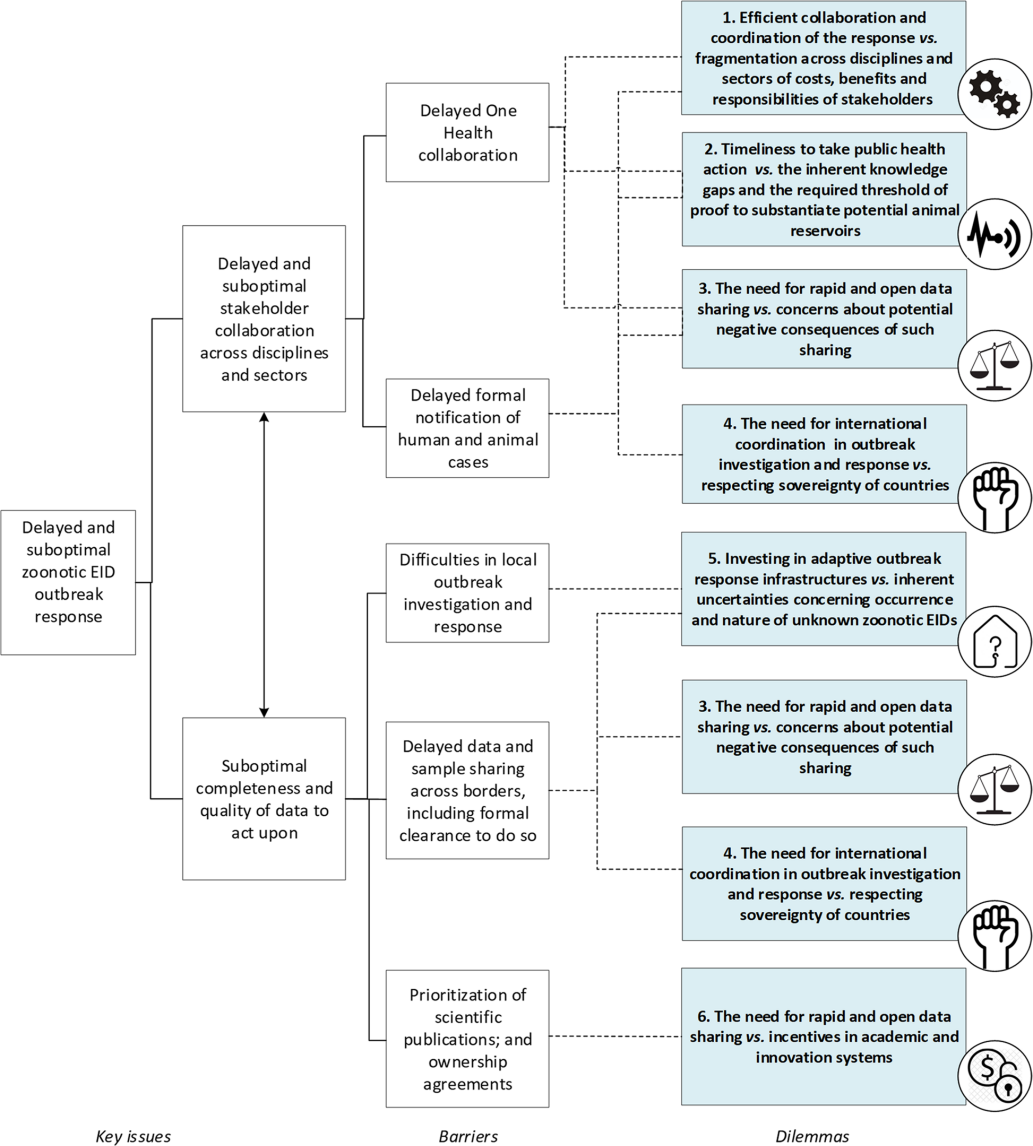
Key issues in data sharing hampering the outbreak response (left) ultimately resulted from six systemic data sharing dilemmas for stakeholders (right, in blue). The barrier Suboptimal public health and lab research collaboration (2) has not been taking into account as it mainly affected national stakeholders, with limited effect on all other stakeholders

Based on the root-cause analysis of the barriers and enablers, and their influence on data sharing for national, international and global stakeholders, two interlinked key challenges in the outbreak response were identified: 1) suboptimal stakeholder collaboration, and 2) delayed sharing of data and samples, with suboptimal quality and completeness, necessary to help stakeholders respond effectively to the outbreak. In Fig. 2 it is shown how the barriers contributed to these challenges (full version with root causes in Supplementary Material 2).

Remarkably, the barriers in data sharing were rooted in—combinations of—the following six core dilemmas:

1. Efficient outbreak response needs collaboration and coordination; but this is hampered by fragmentation across disciplines and sectors of costs, benefits and responsibilities of stakeholders involved;
2. Timeliness to take action in the interest of public health is crucial; yet this is compromised by the need for striking a balance between the inherent knowledge gaps and the required threshold of proof to substantiate potential animal reservoirs. This threshold, while necessary to avoid causing panic and blame, seemed to be higher if the animals are considered invaluable possessions in society, and seem not to be affected;
3. There is a need for rapid and open data sharing during outbreaks; but this is impeded by concerns about potential negative consequences of such sharing reflecting on the individual, institution, and/or country;
4. International assistance and coordination in outbreak investigation and response is necessary, but the sovereignty of countries must also be respected;
5. Investments in adaptive outbreak response infrastructures is required, but this is costly and compromised by inherent uncertainties concerning occurrence and nature of unknown zoonotic EIDs;
6. There is a need for rapid and open data sharing, but this is hindered by incentives in academic and innovation systems.

## Discussion

Using the MERS epidemic as a model, this case study investigated the underlying causes of eight known and new barriers to multi-stakeholder collaboration and sharing data for zoonotic EID outbreak response encompassing different sectors and disciplines, and operating at national, international and global levels. Barriers were found to be highly interconnected as shown by the overlap of underlying causal factors, and were classified in context of six rooted and systemic dilemmas. The unraveling of the barriers into causes and root-causes, as well as placing these into context of six persistent dilemmas, has not been comprehensively described in this context before, complementing existing literature [6, 7, 12].

The consolidated input from the study participants indicated that the current organization of stakeholders and their institutions, as well as the alignment of outbreak investigation and response activities across disciplines and sectors, does not meet society’s needs in terms of swiftness and effectiveness in outbreak response. Rather, it is demanding an enhanced level of coordinated and structural multi-stakeholder collaboration, particularly at the human-animal interface in the early events of a spillover- most notably for notification and reporting, case definition, and case finding and contact tracing—where the most significant delays in the MERS outbreak response were identified [14].

Although the need for integrated One Health collaboration has increasingly been recognized [9, 13, 18], this study identified in the context of a zoonotic EID outbreak a systemic dilemma on (timeliness of) taking action in the interest of public health, balancing inherent knowledge gaps and meeting a required threshold of proof to substantiate an involvement of certain animal reservoirs. It underlines the necessity of early involvement of animal health stakeholders in outbreak preparedness and response, as well as the need for structural One Health collaboration, at the national and global level. This can contribute to timely sharing of pathogen information at the human-animal interface for surveillance, early detection and reporting, supporting the recommendations by the International Health Regulations (IHR) review committee on the proposed amendments to the IHR [19].

Two enablers identified in this context were perceived to have underscored embracing a One Health approach. At the national level, establishing a dedicated One Health Joint field investigation team unifying mandates, activities, etc. among sectors and disciplines, and at the global level, the tripartite WHO, WOAH (former OIE) and FAO collaboration, if initiated early on in the outbreak. Since the MERS epidemic, the tripartite – and now known as the Quadripartite –has been actively working on strengthening global health security at the human-animal interface in support of a One Health approach [20], addressing some of the root causes related to technical infrastructure and guidance on standardized protocols. The recently launched One Health Joint Plan of Action, as well as the establishment of the One Health High-Level Expert Panel (OHHLEP), is expected to further enhance their cross-sectoral collaboration and activities [21, 22]. However, this study indicates that the inequitable division of investments, costs and benefits across disciplines and sectors must also be addressed, for example by a unique funding structure to support the shared responsibility of integrated outbreak preparedness and response [23].

Remarkably, this study showed that the response infrastructures and resources to help contain zoonotic EIDs in a wealthy country like Qatar seemed insufficient to mount an immediate, rapid outbreak investigation and response. The dilemma of investing in an adaptive outbreak response infrastructure for EIDs has been described for Low and Middle Income Countries (LMIC) [24, 25], but it had not yet not been similarly described for well-resourced countries, as they were challenged by one or more zoonotic EIDs. While focusing on investments to build local laboratory capacity is important for improved outbreak preparedness and response, this is also rather requiring sophisticated and advanced infrastructure and expertise, raising this challenge for decision-making on public investments.

In this respect, an interesting result highlighted by this study is the importance of international collaborating partners as external advisors and/or reference centers to help enhance the capacity to rapidly detect, investigate and respond to zoonotic EIDs. In the case of MERS, such collaboration proved useful in rapid validation of suspected cases, and implementation of standardized protocols for data collection and handling, ultimately strengthening the local outbreak investigation and response capacities. The cross-border sharing of and access to outbreak related data should therefore be facilitated. To support this, the root causes related to difficulties in sample sharing across borders (barrier 6), including formal clearance to do so (barrier 5), as well as to stakeholders’ concerns about negative consequences and interests (barriers 5, 7, 8) should be appropriately addressed. Hence, action is needed not only at the national level, but also at global level [19, 26].

The complex level of multi-stakeholder collaboration required in a zoonotic outbreak response, thus means finding a way to overcome the barriers and dilemmas that have been identified to hamper data sharing, and resulting from the current approach where stakeholders operate in different silos (e.g. sectors, disciplines). This has been similarly observed in innovation systems [27, 28], in which a manifold of stakeholders interact, and is characterized by reciprocity and feedback mechanisms, and various system imperfections or failures can hinder innovation. Moreover, while the cooperative activities require divers inputs from the stakeholders, the generated outcomes are not equally beneficial or valuable for each stakeholder [29], further explaining the existence of the dilemmas. An example is the discussion on open sharing of viral genomic data, which is perceived to be more beneficial for high-income countries, while LMIC are likely more prone to the risk of being adversely affected by inequitable consequences from the use of samples and data shared [30]. This study indicates that addressing barriers to data sharing, and stakeholders’ dilemmas, requires comprehensive solutions, considering the complexity and multitude of root causes that underlie them, as well as the scope of their implementation for each of the stakeholders involved.

The identified enablers may shed more light on this approach. The variety and multitude of enablers identified in this study showed the willingness of stakeholders to overcome or circumvent the barriers that hampered data sharing. However, this study also pointed out the inherent trial and error nature of implementing such enablers: not all enablers had similar capacity to enhance data sharing or alleviate barriers*.* Hence, no intervention works for all stakeholders, and their effects may differ according to the context in which they are implemented*.* Furthermore, specific outcomes of interventions cannot be defined a priori but are emergent [31]. As such, no enablers exist as predefined magic bullets. Careful consideration on the scope of their implementation, and monitoring and evaluation of their effect on the barriers and underlying dilemmas, are thus necessary.

In this respect, it is interesting to observe the application of enablers, identified for the MERS epidemic such as the expedited publication process and pre-established collaborations and networks, being more broadly applied during the COVID-19 pandemic. Pre-print sharing of the content of scientific publications (e.g. through BioRxiv) helped to address knowledge gaps inherent to an EID outbreak [5]. Yet, concerns were raised towards the overflow of scientific information (‘infodemic’), and the lack of peer review, in some cases leading to retraction of significant publications, causing confusion amongst public health stakeholders on the action to take [32]. Similarly, pre-established collaborations and networks, supported by (inter)national funders, accelerated outbreak investigation and response studies. However, this has also resulted in increased fragmentation and dispersion of resulting data, as funders have championed different and seemingly competing data sharing initiatives [33], and in an inequity in capacity to generate data, which persisted [34].

To conclude, data access and data sharing during the MERS outbreak investigation and response could have benefitted from a structural and more timely One Health collaboration at the human-animal interface in the early events of a spill-over, taking action in the interest of public health, supported by timely guidance from the WHO, WOAH, and FAO for such a One Health approach, although the Tripartite – and now Quadripartite – have made improvements since the MERS outbreak. While investments in local outbreak investigation infrastructures could have improved the technical and operational capacity, collaboration with international institutes or reference laboratories should also be facilitated to enhance and strengthen the local outbreak investigation and response capacities to rapidly detect, investigate and respond to zoonotic EIDs. This means facilitating international sample and data sharing, by unifying or simplifying regulations to do so, as well as a priori defining, preferably standardized, appropriate reciprocity mechanisms for data sharing, including for academic reward.

This case study has some limitations. First of all, despite key stakeholders from various groups participating in this study, the underrepresentation of stakeholders from Saudi Arabia is noted as a limitation, due to a lack of response and unavailability to participate within the timeframe of this study. Second, as this study investigated stakeholders’ experiences in data sharing during the MERS epidemic starting in 2012, recall bias may have led to over-reporting or underreporting of actual barriers and enablers experienced. Finally, the scope of this study did not allow for analyzing the negative consequences for data sharing by stakeholder groups; nor did it allow for examining the perceptions of the pharmaceutical industry who is deemed influential in the dynamics and interests of data and sample sharing.

Despite these limitations, this comprehensive study deciphered the complexity of data sharing in a multi-stakeholder environment, during a zoonotic EID outbreak response. While improvements to strengthen outbreak investigation and response at the human-animal interface have been made since the MERS epidemic, the root causes deciphered in this study further indicate areas of improvements. In light of the ongoing review process of proposed amendments to the IHR, some of the, so far unpublished, data on barriers and enablers to data sharing in the early stages of the MERS epidemic provided by this study, could provide helpful to that discussion. Finally, this study underlines the importance of necessary dialogue between and amongst relevant stakeholders to achieve a better informed, and more sophisticated decision on the improvement of the data sharing practice through reciprocity and incentive mechanisms, and considering the inherent trial and error nature thereof.

### Supplementary Information

**Additional file 1.** Supplementary Material.

## Data Availability

Full results are provided in Supplementary Materials 1a-d and 2. Materials that were used during data collection (e.g. standardized interview guide) are provided in Supplementary Materials 3 and 4.

## Abbreviations

Barrier: Factor that hampered or delayed data sharing during the MERS outbreak, usually the observed or experienced (visual) problem
Causal factor: The underlying cause of a barrier to data sharing
COVID-19: Coronavirus Disease 2019
Dilemma: Apparent conflicts in interests, norms and values, resulting from the need to work across separate systems and silos (e.g. sectors, disciplines), each having their own interests, norms and values by which the system operates. Together with the root causes, the dilemmas often are the source to find a solution to the barriers
EID: Emerging infectious disease
Enabler: Factor that motivated or facilitated data sharing during the MERS outbreak. Often, it helped to circumvent a certain barrier
FAO: Food and Agriculture Organization of the United Nations
IHR: International Health Regulations
Key issue: Symptoms of the barriers, i.e. the negative effects of the barriers to data sharing on the MERS outbreak response
LMIC: Low and Middle Income Countries
MERS: Middle East respiratory syndrome
MERS-CoV: Middle East respiratory syndrome Coronavirus
MTA: Material Transfer Agreement
MoU: Memorandum of Understanding
One Health: The term One Health refers to an integrated, unifying approach to balance and optimize the health of people, animals, and the environment, in particular relevant for prevention, detection and response to zoonotic EID outbreaks
OHHLEP: The One Health High-Level Expert Panel has an advisory role to the Partners of the Quadripartite to support their provision of evidence-based scientific and policy advice to address the challenges raised by One Health
OIE: Former World Organization for Animal Health, now WOAH
Quadripartite: The Tripartite partnership for One Health brought together the WHO, the FAO, and the WOAH, and with the inclusion of the UNEP, it has more recently become the Quadripartite, with the aim to develop and implement a joint plan of action to fight zoonotic and antimicrobial resistance threats
Root cause: The core issue to a barrier and the highest-level underlying cause that can be identified, often systemic in nature, that lead to a cascade of causes-and-effects that ultimately leads to the barrier
Tripartite: See Quadripartite above
UNEP: United Nations Environment Programme
WOAH: World Organization for Animal Health
WHO: World Health Organization
