# Supplementary material for: Six dilemmas for stakeholders inherently affecting data sharing during a zoonotic (re-)emerging infectious disease outbreak response
Source: BMC Infect Dis. 2024 Feb 12;24:185. doi: 10.1186/s12879-024-09054-0 (PMC10863217; doi:10.1186/s12879-024-09054-0)
Supplement: Supplementary file 1 — Additional file 1. Supplementary Material. [file 12879_2024_9054_MOESM1_ESM.docx]

# Supplementary Material

# Causal trees of the eight barriers, with enablers

a. Causal analysis of the two barriers related to stakeholder collaboration across disciplines and sectors, of which prominent One Health (barrier 1). Root causes predominantly revealed fragmented responsibilities and priorities of stakeholders, resulting in delayed stakeholders’ sharing of data, collaboration and alignment of response activities across disciplines and sectors. From left to right, first column represents the barriers as experienced by the key stakeholders, middle columns represent the causal factors, and the last column represents the root causes for the existing barriers, where lines indicate the causal argumentation. The enablers are shown as rounded rectangles according to the causes which these enablers predominantly addressed or reinforced (green refers to indisputable enablers; blue refers to situational enablers; orange refers to tentative enablers).


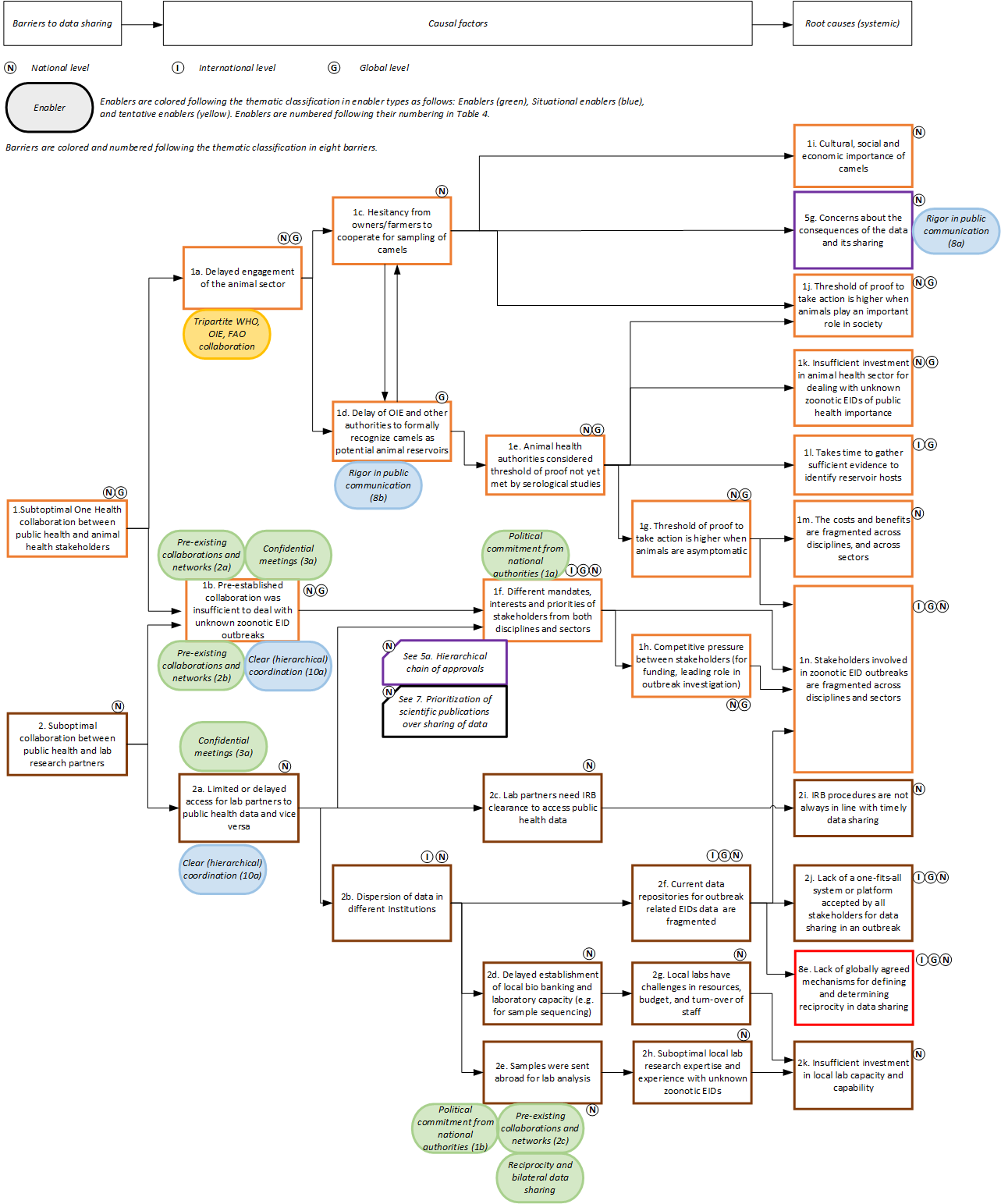


b. Causal analysis of the barrier that was more technical in nature. Root causes related to the technical and operational capacity and capability to collect, generate and share data as part of a routine, complex outbreak response at the human-animal interface, but also to fragmentation issues in stakeholder collaborations. From left to right, first column represents the barriers as experienced by the key stakeholders, middle columns represent the causal factors, and the last column represents the root causes for the existing barriers, where lines indicate the causal argumentation. The enablers are shown as rounded rectangles according to the causes which these enablers predominantly addressed or reinforced (green refers to indisputable enablers; blue refers to situational enablers; orange refers to tentative enablers).


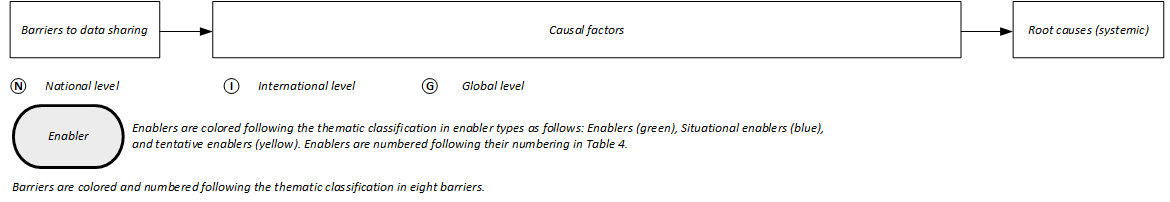

c. Causal analysis of the barrier that related to compliance to the regulatory framework. Perhaps the most complicated barrier in terms of the variety in root causes since causal trees from several other barriers (1,3 and 5) affected the delay in formal notification, in addition to barrier specific root causes. Root causes not only related to the formal notification process, but also to technical issues, concerns about negative consequences, and fragmentation issues in stakeholder collaborations. From left to right, first column represents the barriers as experienced by the key stakeholders, middle columns represent the causal factors, and the last column represents the root causes for the existing barriers, where lines indicate the causal argumentation. The enablers are shown as rounded rectangles according to the causes which these enablers predominantly addressed or reinforced (green refers to indisputable enablers; blue refers to situational enablers; orange refers to tentative enablers).


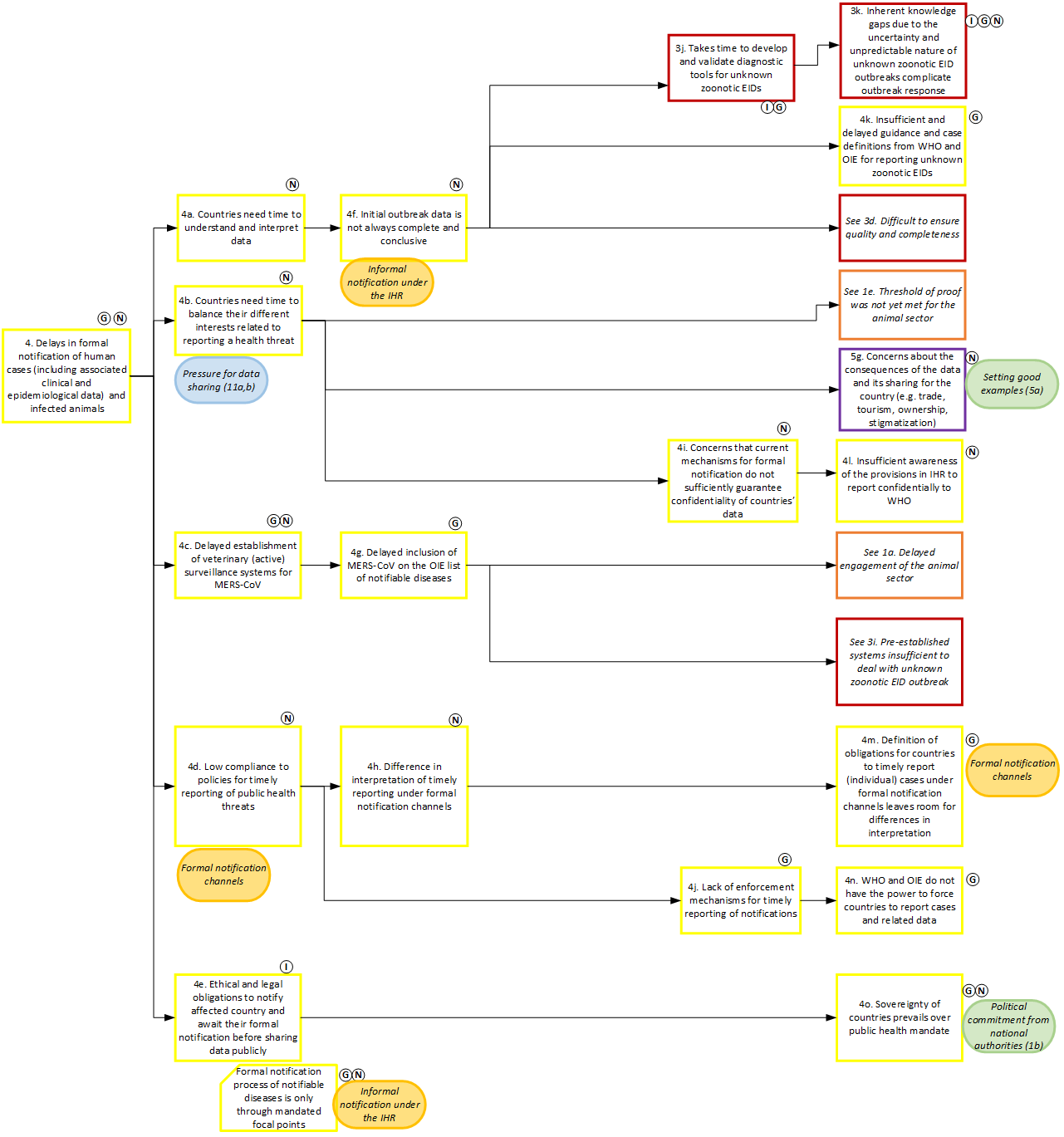

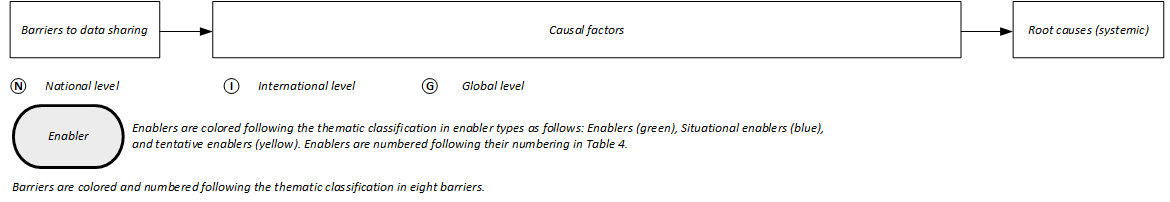


d. Causal analysis of the two other barriers related to compliance to regulations and authorization for sharing. Root causes related to concerns about negative consequences of data sharing, complexity in regulations governing international data sharing, as well as technical difficulties. From left to right, first column represents the barriers as experienced by the key stakeholders, middle columns represent the causal factors, and the last column represents the root causes for the existing barriers, where lines indicate the causal argumentation. The enablers are shown as rounded rectangles according to the causes which these enablers predominantly addressed or reinforced (green refers to indisputable enablers; blue refers to situational enablers; orange refers to tentative enablers).


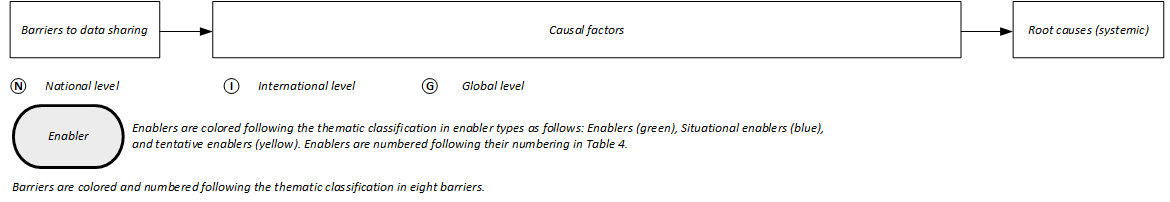

e. Causal analysis of two barriers that related to ownership rights, (self)interests of stakeholders, and insufficient incentives for data sharing. Causal trees related to motivational arguments as well as concerns about the lack of reciprocity of data sharing, including the lack of globally agreed and standardized mechanisms for ownership and reciprocity of data sharing during outbreaks. From left to right, first column represents the barriers as experienced by the key stakeholders, middle columns represent the causal factors, and the last column represents the root causes for the existing barriers, where lines indicate the causal argumentation. The enablers are shown as rounded rectangles according to the causes which these enablers predominantly addressed or reinforced (green refers to indisputable enablers; blue refers to situational enablers; orange refers to tentative enablers).


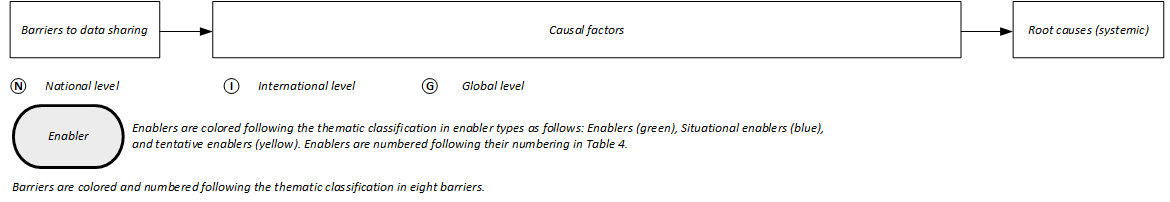


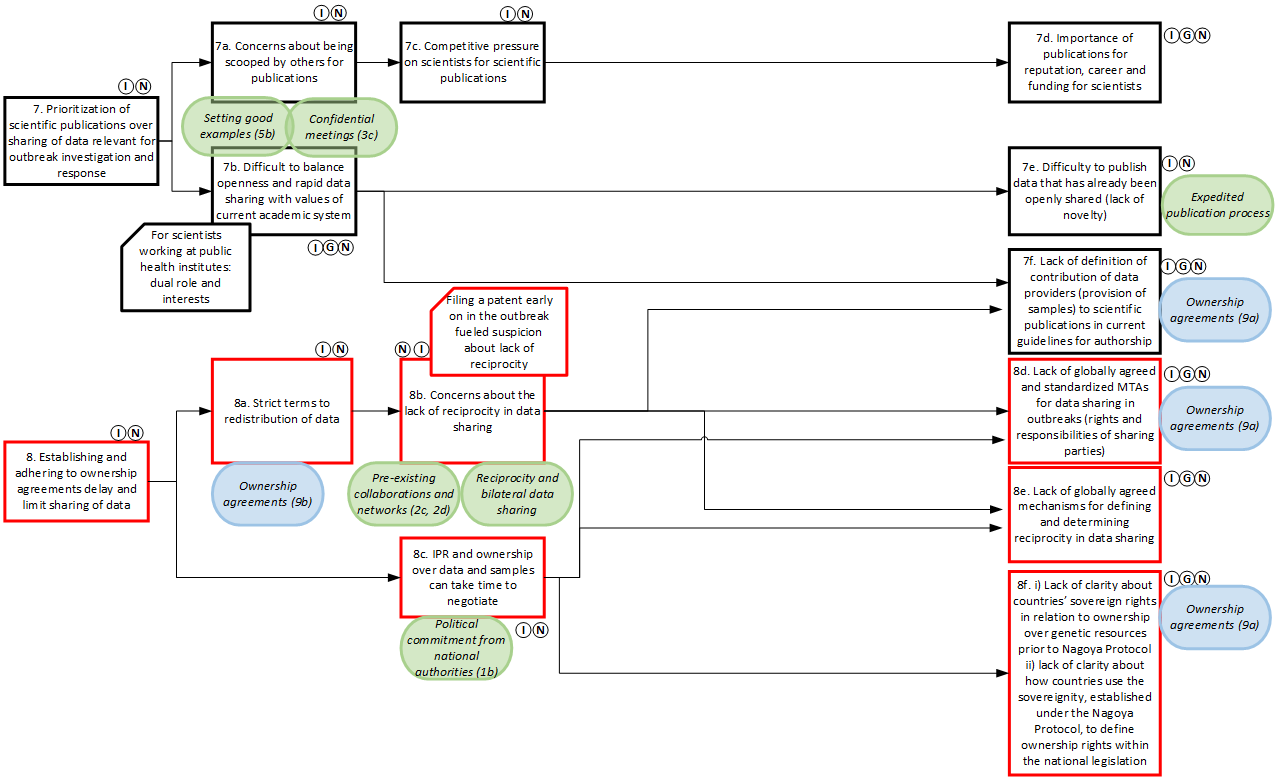


# Key issues in data sharing showed six rooted and systemic dilemmas for stakeholders


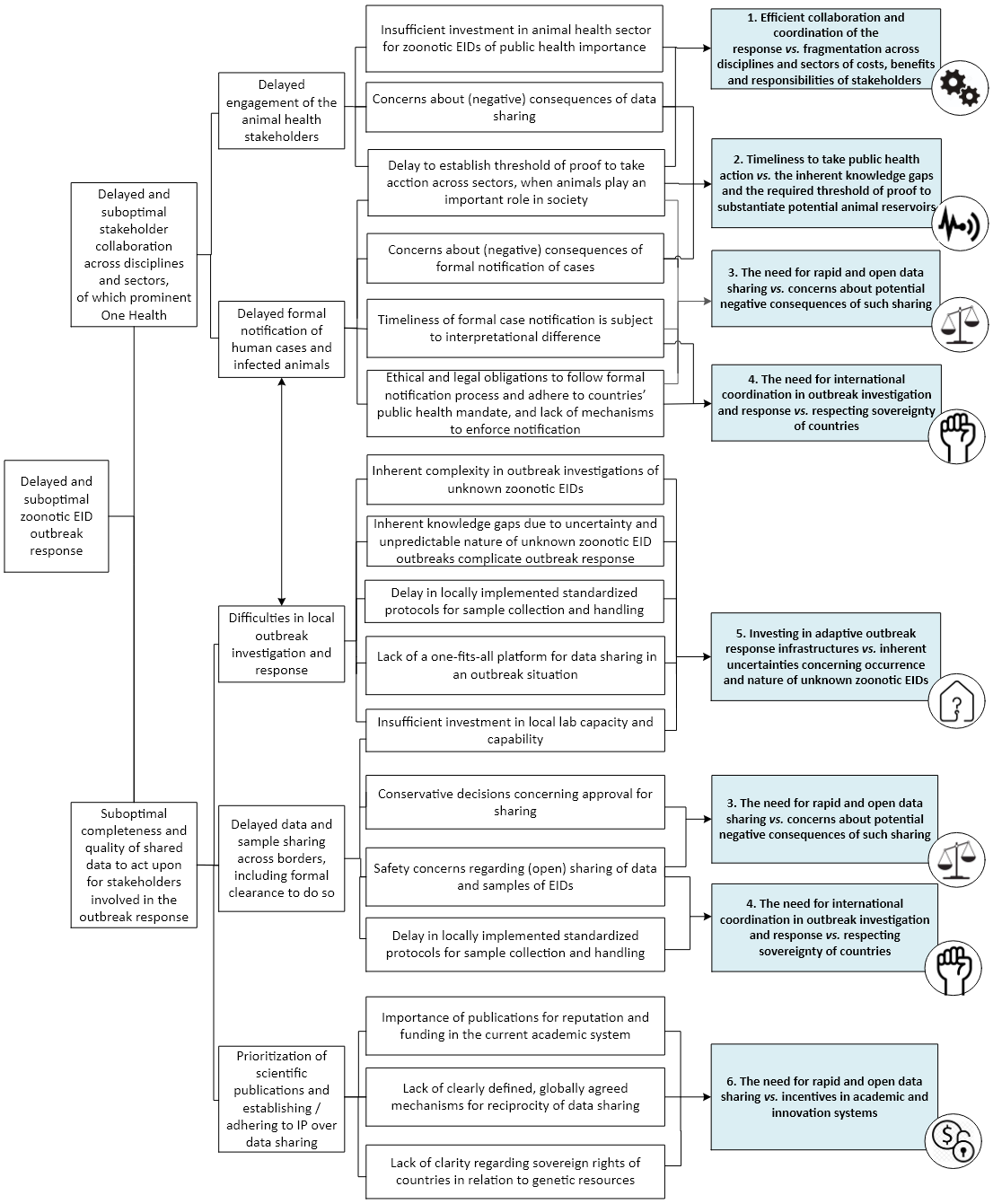


# List with definition of key data and the most commonly used data sharing mechanisms (information sent to the key stakeholders*)

** Using the WHO policy statement on data sharing in the context of public health emergencies as guidance document, this list of definitions for key data was developed from different types of data related to outbreak response or outbreak related research (WHO, 2016), including a list for the most-commonly used open and closed data sharing mechanisms. This comprehensive, yet not conclusive, list of examples was sent to the key stakeholders before the interviews to provide clarity on the focus of this case study.*

## Scope of the SHARE case study: definition of data

The study will focus on the sharing of clinical, epidemiological, and laboratory data as part of the clinical and laboratory research preparedness and response to infectious disease outbreaks. This scope encompasses outbreak research involving:

- Epidemiological investigation and surveillance: tracking of cases and contacts, outbreak investigation, including identification of sources and transmission modes;
- Clinical research: research involving a substantial amount of work related to the observation of, data collection from, or diagnostic or therapeutic intervention on multiple or individual patients;
- Laboratory research: research involving all activities concerning laboratory outbreak response and research, including the sharing of microbial genetic resources, i.e. strains and genetic sequence data from pathogens and related metadata, samples, assays, protocols, and experiences.

The following types of data that encompasses above specified clinical, epidemiological and laboratory research will be included in this case study. These types of data are categorized into three categories based on different considerations for data sharing in public health emergences in each of the categories, using the WHO policy statement on data sharing in the context of public health emergencies as guidance document.

1. ***Data related to and/or necessary for outbreak surveillance, epidemiology and emergency public health response***

- **Epidemiological investigation data - human**:
  - Suspected and/or confirmed case notifications
  - Epidemiological evidence of human-to-human transmission (contact tracing)
  - Estimates of duration of infectivity and incubation period
  - Suspected and/or confirmed sources
  - …
- **Epidemiological investigation data - animal**:
  - Suspected and/or confirmed case notifications of infection
  - …
- **Laboratory/ clinical diagnostic data**:
  - Case definitions of infections, i.e. diagnostic analyses of samples
  - Diagnostics assays and related materials (e.g. reagents) for use
  - Serological data from surveys to assess the extent of the outbreak
  - …
- **Information related to epidemiological investigation and surveillance as part of the public health response**:
  - WHO guidelines for surveillance, case definition and laboratory testing, case investigation, and infection control
  - OIE guidelines for case definition
  - Clinical guidelines for treatment of cases
  - Scientific/ evidence based protocols for sampling and diagnosis
  - (National) outbreak response plan/ (experienced) public health measures
  - National and WHO guidelines on the laboratory safety
  - …

1. ***Genetic sequences and biological samples***
   - Pathogen genomic data
   - Samples from patient and/or suspected cases
   - Samples from suspected infected animals
   - …
2. ***Data related to/ necessary for outbreak related research***

- **Clinical/ epidemiology research data - human**:
  - Patient statistics (related metadata): sex, age, height, weight, disease history and comorbidities, place of residence at time of start symptoms
  - Clinical observational data: natural history of disease and clinical presentation (clinical course of infection, including disease symptoms and disease outcome), as well as in relation to treatment (including but not limited to therapeutic intervention)
  - Clinical trial data: data related to the conduct of clinical trials of diagnostics, therapeutics and preventives such as vaccines
  - …
- **Epidemiology research data – animals**:
  - Epidemiological observational data: natural history of infection, as well as in relation to treatment (including but not limited to therapeutic intervention)
  - …
- **Laboratory research data**:
  - Novel diagnostics for both human and animal populations and related materials (e.g. reagents) for use
  - Cross-validation of diagnostics (already in use and in development)
  - Data on pathogen transmission: source attribution (main animal reservoir and source of zoonotic transmission to humans), dynamics of transmission events (e.g. routes of exposure), geographic distribution, epidemic potential and virulence
  - Data on pathogen-host interaction: correlates of protection, predisposing factors for infection and disease, incubation period, immune-pathogenesis, virulence, viral tropism, host susceptibility, structure-function relationships, markers for disease or infection, viral evolution, phenotypic correlates of viral sequences, pathological and/or histological analyses data of diseased patients and/or animals
  - Relevant (small) animal models for pre-clinical studies
  - Data on pre-clinical studies on effect of treatment options (*in vitro*, immunogenicity, efficacy)
  - Novel vaccines and therapeutics, and (WHO) standards/reference reagents
  - ...

The above defined list of data types is not a conclusive list, as is indicated by the dotted lines. During the case study, types of data can be added to this list that appeared to have been crucial for the outbreak/public health response or for outbreak related research. It is rather meant to provide clarity on the focus of this case study, especially for the interviews with stakeholders.

## Mechanisms of data sharing

The scope of mechanisms of data sharing included in this case study will be as broad and inclusive as possible, yet we will define most commonly used mechanisms of data sharing for the sake of clarity, which will especially be useful for the interviews with stakeholders. It will include formal and informal mechanisms for data sharing. It is roughly divided into open sharing mechanisms and closed sharing mechanisms:

- **Open sharing mechanisms**:
  - Public databases (genomic/ structural/ trial registries)
  - Articles in journals and supplementary material in journals
  - Journal pre-prints
  - Disease specific curated repositories (Nextstrain)
  - General purpose academic repositories (Figshare, Dryad)
  - Big Data approaches (which collate information from public sources; WHO Global outbreak alert and response network/ Global Public Health Intelligence Network, ProMed, HealthMap)
  - Public/online notifications, guidelines and reports (ProMed, WHO and OIE meeting reports and online updates)
  - (Popular) press, media
- **Closed sharing mechanisms**:
  - Institutional repositories (can sometimes be open)
  - Closed consortia
  - Informal professional networks
  - Notifications to WHO and OIE
  - Closed meetings/ missions (e.g. if requirements for making the information available to States parties are not yet fulfilled)

# Interview topic list

## Introduction

Based on your experience / the experience of your institute as… (*what we know about his/her involvement with MERS-CoV*)… during the MER-CoV epidemic (in Qatar); we are contacting you to learn about your experience and opinion on the sharing of data during the epidemic. Before starting we would like to highlight that this interview will be performed according to the Chatham House rules, therefore the information provided will be kept anonymous. We would like to ask your consent to record this interview for further analysis and use, anonymously, in future publications. Is there any point that you would like to clarify before starting the interview?

## Questions

### 1. Confirming stakeholder role and contribution

1. In the invitation letter to this interview, we have described, based on the desk study, how we identified your involvement with the MERS-CoV research/response. Could you shortly confirm or correct your role and task to that, especially in regard to your participation/collaboration with the MERS-CoV research/response in Qatar?

### 2. Description of data sharing practices

Introduce and explain the interview data matrix (Annex 3) and use it as an active tool during the following questions:

1. What are your general feelings about the data sharing practices during the MERS-CoV epidemic? From your experience, during the course of the epidemic, do you think sharing was overall satisfactory on unsatisfactory?
2. Which types of data were typically shared with you and from whom were they coming? Did this differ in the different phases/periods of the epidemic (see the timeline)?

- Points for confirmation: how he/she obtained the data and how the data was used

1. Which type of data were typically shared by you and towards whom did you share these? Did this differ in the different phases/periods of the outbreak (see the timeline)?

- Points for confirmation how he/she shared the data and why he/she shared (perceived importance of the data)

1. Did you - generally speaking and as far as you can see - notice any change in attitude towards data sharing over time, or did you yourself change or had to change your data sharing practices or policy over time? If any changes, can you elaborate on the perceived or suspected motives?

### 3. Enablers to data sharing

1. You have just described some events during the epidemic in which either you shared data or data was shared with you. In your opinion, what were the facilitators, or in other words the enablers, that helped or improved the sharing process the most?

- By enablers we mean any factor that facilitates or motivates data sharing. Those can be conditions, procedures, attitudes, persons, infrastructures, technologies, etc.

1. Depending on one’s position in an organization one might have different views on the possibilities for influencing data sharing. From your personal opinion, are you or your organization in a position – formally or informally – to enforce or facilitate the sharing of outbreak-related data?

### 4. Problems and complications / Barriers to data sharing

1. Changing the topic from enablers to data sharing to possible complications and barriers. From the data that you described was shared with you, did you encounter any serious issues, that did endanger or delay, or could have endangered, the application/use of the data for the epidemic response and control? Concerning:

- The quality or completeness of data?
- The timeliness of sharing?
- Differences on perceptions (the perceived urgency)?
- Procedures or authorization of the sharing?
- (Interpretation of) the legal framework regarding national/international sharing?
- Any other?

1. To make sure that we have a complete overview of possible experienced complications to the sharing of data we ask all interviewees specifically : was there for you at any point in time a need to access specific data that was not available or accessible for you; and which was not yet mentioned in this interview? If so, what type of data was it and why do you think it happened?

- Did you finally managed to overcome this barrier and have had access to the data? How did you do this and how long did it take? How the data would have contributed to the epidemic research/response?

1. Now changing the perspective from receiver of data to provider, have you been in the situation that you or your organization were/was reluctant to share data or had to decide not to share? Was this temporally or definitely? Can you elaborate on the motives?

- Did you find a way to overcome these barriers and if so, how?

### 6. Proposed changes and recommendations

1. We do not finish this interview, without giving you the opportunity to express your final reflections on the experience with data sharing during the MERS-CoV epidemic. In this regard, have you identified any lessons learned (regarding data sharing) that could be useful for future epidemics? Which advices can you give to others in similar/future situations?
2. We all know that there are no clear/easy solutions to address all data sharing challenges. But, do you see any (other) possible solutions or what would be a first step forward in solving these challenges?

- Point for confirmation: who could/should work towards developing/implementing these suggestions? Who should take up the responsibility?

## **Conclusion**

Thank you very much for your time. Your contribution is very important for our research. Please let us know if you would like to be contacted again in the future to provide our preliminary results for feedback and validation.
